# The Impact of Resident Involvement on Surgery for Pelvic Organ Prolapse

**DOI:** 10.7759/cureus.110619

**Published:** 2026-06-10

**Authors:** Andreina Dones, Angela S Yuan, Nani P Moss, Jonathan P Shepherd

**Affiliations:** 1 Department of Obstetrics and Gynecology, University of Connecticut School of Medicine, Farmington, USA; 2 Department of Obstetrics and Gynecology, Trinity Health of New England, Hartford, USA

**Keywords:** laparoscopic surgery, pelvic organ prolapse, resident training, surgical complications, uro- gynaecology

## Abstract

Introduction

Prior research shows that when urogynecologists perform surgery with fellows, operative time increases, without increased adverse events/prolapse recurrence. Outside fellowship training programs, urogynecologists operate with obstetrician/gynecologist (OB/GYN) residents (RES) or physician assistants (PAs). We compared operative time and complications when urogynecologists operate with RES versus PA. We hypothesized that attendings operating with RES would increase operative times without increasing adverse events.

Methods

This retrospective cohort study included all apical pelvic organ prolapse (POP) repairs performed between June 2016 and July 2020 by a single fellowship-trained urogynecologist at two community hospitals, with comparisons between RES and PAs.

Results

We identified 108 POP repairs, with 77 (71.3%) performed with RES assistants and 31 (28.7%) with PAs. RES cases had longer operative times than PA cases (187.5 ± 80.3 vs. 155.7 ± 53.2 minutes, p = 0.045). After adjusting for surgical route, BMI, concomitant hysterectomy, and midurethral sling placement, RES cases remained 49.0 minutes longer (p < 0.001). Complication rates were similar on univariable analysis (24.7% vs. 9.7%, p = 0.11). Urinary tract infections (UTIs) accounted for most complications (12/22, 54%) and did not differ by assistant type (13.0% RES vs. 6.5% PA, p = 0.503). When UTIs were excluded, there were no differences in composite complications (14.5% vs. 3.2%, p = 0.173), though the low number of events limited regression analysis. RES cases also had greater estimated blood loss (64.4 ± 66.4 vs. 33.2 ± 20.6 mL, p < 0.001).

Conclusions

When an expert surgeon operated with a resident rather than a PA, operative time was 49 minutes longer (approximately 30% longer overall). However, complication rates did not increase.

## Introduction

Apical pelvic organ prolapse (POP) is a prevalent condition associated with aging, parity, vaginal delivery, obesity, and menopausal status [1-2]. It is projected that the number of women with POP will rise by 46%, from 3.3 million to 4.9 million, between 2010 and 2050 [3]. Given this increasing prevalence, a greater need for surgical repair is anticipated. Multiple studies demonstrate that surgical correction of POP is safe, with low complication rates and high patient satisfaction, making it a widely accepted treatment option for patients [4-8].

All residency programs require residents (RES) to rotate through urogynecology to learn surgical techniques involved in prolapse repair. Residency programs with a urogynecology fellowship program inherently prioritize fellow training on the urogynecology-specific portions of these procedures, but in programs without a fellowship program, the residents are more often the first assistant. Carter-Brooks et al. conducted a large retrospective study in 2017 of 208 robotic-assisted sacrocolpopexy (RASC) cases, comparing outcomes when an expert attending operated with a physician assistant (PA) versus a fellow. Fellow involvement increased operative time by 34 minutes but did not affect prolapse recurrence or complication rates [9]. We expand on this prior work by investigating the impact on operative times and complications when an expert attending operates with RES versus PAs when no fellows are involved. We hypothesize that the time differences with a resident will be more marked than those previously observed with fellows.

Current literature suggests that RES involvement may increase operative times without increasing complications. One study found that a trainee group (fellows and surgeons with fewer than 30 laparoscopic sacrocolpopexy (L/S ASC) cases) had longer operative times but no increase in adverse events [10]. Another study of 20 RASC cases performed with an attending surgeon and 21 cases assisted by fourth- and fifth-year residents found no difference in operative time or complications after adjusting for concomitant procedures [11]. However, both studies included senior residents and fellows, which may limit the generalizability of their findings. Overall, there are limited data specifically evaluating the impact of third- and fourth-year gynecologic residents as first assistants on surgical outcomes. 

There is increasing pressure on academic physicians to be both highly productive in the operating room (OR) and to train future surgeons, two goals that are often in direct competition. As academic physician compensation increasingly shifts toward models tied to clinical productivity, it is important to understand the impact of resident surgical training on OR efficiency, so that this essential but time-intensive responsibility, which is central to an academic hospital’s mission, can be appropriately recognized and incentivized. Therefore, the objective of this study is to determine whether an expert surgeon operating with third- and fourth-year residents, compared with a PA, affects operative time and complication rates in apical prolapse repairs. We hypothesize that cases involving RES will have longer operative times without an increase in complications.

This article was previously presented as a poster at the annual American Urogynecological Society’s Pelvic Floor Dysfunction Week, 2024, held in Washington, DC, October 22-25, 2024.

## Materials and methods

Study design

This study followed a similar protocol to the prior study by Carter-Brooks et al., which examined fellow involvement [9]. We used a retrospective cohort design including women aged 18 years and older who underwent apical prolapse repair between June 2016 and July 2020 by a fellowship-trained FPMRS surgeon at two community hospitals. The cohort was identified using a surgical calendar and included all women who underwent apical prolapse repair using procedures such as laparoscopic sacrocolpopexy (L/S ASC), laparoscopic uterosacral ligament suspension (L/S USLS), vaginal USLS, vaginal sacrospinous ligament suspension (SSLS), or colpocleisis.

Data collection

Data were collected retrospectively from the electronic medical record (EMR). EMRs were manually reviewed for demographic information, past medical history, and examination findings, including age, race, marital status, parity, BMI, menopausal status, smoking status, medical comorbidities, previous abdominal and pelvic surgeries, and preoperative POP quantification (POP-Q) measurements [12]. In addition, estimated blood loss (EBL), length of stay, intraoperative and hospital complications, and concomitant surgical procedures were collected. Lastly, EMRs were reviewed for postoperative complications (within six weeks of surgery). All cases in the cohort were included in the analysis. 

The apical prolapse repair cases were categorized based on whether the expert surgeon operated with a RES or a PA. Assignment to RES versus PA assistance was determined by hospital location and was not influenced by case complexity or other clinical factors. All cases at one hospital involved third- or fourth-year residents, representing their first exposure to urogynecology. In contrast, because no residents were present at the other community hospital, all cases there were performed with a single PA with more than 15 years of experience in assisting gynecologic surgeries. The PA participated throughout the entire procedure. When RES assistants were involved, they performed portions of the operation appropriate to their skill level as determined by the attending surgeon.

When a uterus was present, concomitant hysterectomy was performed with oophorectomy and/or salpingectomy performed at the surgeon's discretion based on age and family history. Additional procedures, such as mid-urethral sling (MUS), posterior repair, anterior repair, and lysis of adhesions, were performed as indicated. Prolapse repair approaches include L/S ASC, L/S USLS, vaginal USLS, vaginal SSLS, and colpocleisis. The approach to prolapse repair was decided preoperatively at the attending surgeon’s discretion during office assessment. 

The primary outcome, operative time, was defined as the interval from skin incision to skin closure, measured in minutes. Secondary outcomes included surgical complications. All complications identified through chart review were recorded through six weeks postoperatively. Complications were analyzed as a composite outcome, defined as positive if any of the following occurred: bowel injury, bladder or ureteral injury, transfusion, conversion to laparotomy, sacrocolpopexy mesh exposure, readmission, or postoperative infection. Postoperative infections included surgical site infections and urinary tract infections (UTIs). We acknowledge that UTIs are common after POP surgery, with reported rates of 27.3% [13]. However, they generally have low morbidity and minimal long-term consequences. Therefore, complications were analyzed in two ways: including and excluding UTIs as a complication. 

Statistical analysis

The study population was a convenience sample of patients treated during the defined study period at two hospitals, resulting in an approximate 2:1 distribution of cases with resident assistants compared with PA assistants. The prior study on which this analysis was based enrolled 208 patients and demonstrated a 31-minute difference in operative time (p < 0.001) [9]. Based on these findings, we estimated that a sample size of approximately 100 patients would be sufficient, given the anticipated larger effect size with residents compared with fellows. Ultimately, we observed a significant difference, indicating that the study was inherently adequately powered. 

Patient demographics, medical comorbidities, and surgical factors were compared in cases performed with a RES vs. those performed with a PA. Chi-squared, Fisher's exact, and t-tests were used to assess baseline differences. Results are presented as mean ± standard deviation (SD) for continuous normally distributed variables, median with interquartile range (IQR) for continuous variables not normally distributed, and frequency (percentages) for categorical variables. Linear regression models were used to identify factors that had an impact on operative time, and results are presented as beta coefficients (minutes) with a p-value. 

To avoid overfitting the model, we combined all vaginal cases and all laparoscopic cases into two groups for time comparison, as this was considered the most appropriate grouping given their similar operative durations. The alternative approach of including all five procedure categories was not statistically feasible. Variables with p < 0.2 on univariate analysis were considered candidate predictors for multivariable regression models, which were constructed using backward elimination and verified with forward selection methods. Data analysis was performed using SPSS Statistics (IBM Corp., Armonk, NY). We also planned to perform logistic regression to evaluate the effect of RES vs. PA on complications while adjusting for confounders. However, low rates of complications precluded this analysis.

## Results

Patient characteristics

Of the 108 apical prolapse repairs, 77 (71.3%) were performed with a RES and 31 (28.7%) with a PA. The case breakdown did not differ between RES and PA and included 36 (33.3%) L/S ASC, five (4.6%) L/S USLS, 31 (28.7%) vaginal USLS, 15 (13.9%) vaginal SSLS, and 21 (19.4%) colpocleisis. Demographic and other preoperative variables were also similar between groups (Table 1, all p > 0.05). Patients were predominantly White (n = 80, 78.4%) and postmenopausal (n = 87, 82.1%), with a mean age of 60.43 ± 13.69 years and a BMI of 28.6 ± 6.2 kg/m². The majority of patients were ASA Class 2 (n = 78, 72.2%), and 12 (11.2%) were tobacco users. Preoperative prolapse stage, based on POP-Q, was most commonly stage II (n = 51, 47.7%) or stage III (n = 54, 50.5%). A total of 38 (35.2%) patients had undergone a prior hysterectomy.

**Table 1:** Baseline characteristics ^a^Continuous data are presented as mean ± SD. ^b^Categorical data are presented as frequency (percentage) BMI: body mass index; SD: standard deviation

Characteristics	Resident (n = 77)	Expert surgeon (n = 31)	P-value
Age^a^, years	61.8 ± 14.9	58.8 ± 9.9	0.360
Parity^a^	2.9 ± 1.4	2.5± 1.1	0.205
Race^b^	--	--	--
Caucasian	58 (77.3)	22 (88.5)	0.788
Other	17 (22.7)	5 (18.5)	--
BMI^a^, kg/m^2^	28.4 ± 6.5	29.3 ± 5.3	0.483
Postmenopausal women^b^	63 (84.0)	24 (77.4)	0.418
Past medical history^b^	--	--	--
Diabetes	9 (11.7)	2 (6.7)	0.724
Hypertension	41 (54.7)	20 (64.5)	0.394
Coronary artery disease	5 (6.5)	4 (12.9)	0.275
Current smoker	9 (11.7)	3 (10.0)	1.000
Past surgical history^b^	--	--	--
Abdominal or pelvic surgery	57 (74.0)	18 (58.1)	0.113
Hysterectomy	27 (35.1)	11 (35.5)	1.000
Midurethral sling	4 (5.2)	2 (6.5)	1.000
Prolapse surgery	7 (9.1)	6 (19.4)	0.190
Preoperative prolapse stage^b^	--	--	0.652
Stage II	37 (48.1)	14 (46.7)	--
Stage III	38 (49.4)	16 (53.3)	--
Stage IV	2 (2.6)	0 (0)	--

Surgical factors 

Surgical outcomes are summarized in Table 2. There was no difference between the groups in concomitant procedures, with similar rates of concomitant hysterectomy (62.3% RES vs. 64.5% PA, p = 1.00) and midurethral slings (5.2% RES vs. 12.9% PA, p = 0.223).

**Table 2:** Surgical and postoperative factors ^a^Catergorical data are presented as frequency (percentage). ^b^Continuous data are presented as mean ± SD. ^c^Defined as any one or more of the following: bladder injury, bowel injury, conversion to laparotomy, transfusion, small bowel obstruction/ileus, sacrocolpoexy mesh exposure, post-operative infection EBL: estimated blood loss; UTI: urinary tract infection; SD: standard deviation

Factors	Resident (n = 77)	Expert surgeon (n = 31)	P-value
Concomitant surgery^a^	--	--	--
Hysterectomy	48 (62.3)	20 (64.5)	1.000
Midurethral sling	4 (5.2)	4 (12.9)	0.223
Lysis of adhesions	5 (6.5)	1 (3.2)	0.671
Perineorrhaphy	30 (39.0)	11 (35.5)	0.828
Anterior repair	11 (14.3)	2 (6.5)	0.340
EBL^b^, mL	64.3 ± 66.4	33.2 ± 20.6	< 0.01
Operative time^b^, min	187.5 ± 80.3	155.6 ± 53.2	0.045
Any complications^a, c^	19 (25)	3 (9.7)	0.112
UTIs	10 (13)	2 (6.5)	0.503
Bladder injury	1 (100)	0 (0)	1.000
Bowel injury	2 (100)	0 (0)	1.000
Conversion to laparotomy	4 (100)	0 (0)	0.323
Length of stay^a^, hours	30.8 ± 28.5	33.2 ± 20.6	0.560
Readmission^a^	0 (0)	0 (0)	N/A
Mesh exposure^a^	0 (0)	0 (0)	N/A

Analysis of operative time

Before adjustment for confounders, operative time was significantly longer in cases with RES (187.5 ± 80.3 vs. 155.6 ± 53.2, p = 0.045). Blood loss was significantly higher in RES cases (64.4 ± 66.4 vs. 33.2 ± 20.6 mL, p < 0.001). Linear regression was then performed to control for covariates that may have impacted operative time (Table 3). After adjusting for surgical approach (vaginal vs. laparoscopic), BMI, concomitant hysterectomy, and concomitant midurethral sling, the RES group was associated with an increase in operative time of 49.04 minutes (p < 0.001). Vaginal surgeries (USLS, SSLS, and colpocleisis) were shorter than laparoscopic procedures (ASC and USLS) by 62.6 minutes (p < 0.001, Table 3). Cases were also longer with increasing BMI and with concomitant hysterectomy and midurethral sling.

**Table 3:** Operative time linear regression Positive β-values signify an increase in operative time (measured in minutes), and negative β-values signify a time decrease

Covariates	Unadjusted			Adjusted	
	β (mins)	P-value		β (mins)	P-value
Constant	N/A		N/A	155.73	N/A
Resident as first assistant	31.86		0.045	49.04	< 0.001
Vaginal approach (compared to the laparoscopic approach)	-48.48		< 0.001	-62.56	< 0.001
Body mass index (per unit)	2.37		0.043	3.13	0.002
Concomitant hysterectomy	66.60		< 0.001	71.84	< 0.001
Concomitant midurethral sling	42.68		0.121	59.10	0.007

Analysis of complications 

Complications occurred in 22 patients, with no difference identified on univariable analysis between RES and PA (24.7% vs. 9.7%, p = 0.11, Table 2). The most common complication was postoperative UTI in 12 patients (11.1%), which accounted for 54% of total complications (Table 2). There were 10 UTIs in the RES group (13.0%) and two (6.5%) in the PA group (p = 0.503). When UTIs were excluded, there was no difference in the rate of other composite complications between groups (14.5% RES vs. 3.2% PA, p = 0.173). Given that a convenience sample was used, a post hoc power calculation showed that, using these complication rates and assuming a similar ratio of RES to PA cases, a total of 373 patients would have been required to detect a statistically significant difference in complications. Individual complications were each very rare and were also underpowered to detect differences between RES and PA (all p > 0.05). There was one (1.3%) bladder injury, two (2.6%) bowel injuries, and four (5.2%) conversions to laparotomy, all occurring in cases in which RES was used. The only non-UTI complication in the PA group was the inability to visualize ureteral efflux at the completion of the case, which resolved after ureteral stents were left in place overnight. There were no mesh exposures in either group.

## Discussion

Our findings suggest that residents negatively impact OR efficiency but do not increase complications. An expert surgeon operating with a resident instead of a PA had an increase in operative time of 49.04 minutes (approximately 30% longer). This is consistent with previous work by Carter-Brooks et al., in which a fellow, who has more surgical experience than a resident, increased operative time by 34 minutes compared to a PA [9]. Other studies also support that trainee involvement in POP repair is associated with longer operating times [10,11,14,15,16]. We hypothesize that the increased operative times may be attributable to early experience in the learning curve. After multiple cases, residents will likely require less instruction and demonstrate improved surgical technique. A few studies have found that after approximately 15 to 30 cases, operating times decrease [17-19].

In addition, our study found that trainees do not increase the risk of complications. We found that postoperative complications, other than UTI, were rare, which is reassuring for patients but limited our ability to adjust for confounders. We did find that RES cases had higher estimated blood loss, but the additional 31.2 mL is of uncertain clinical significance. There is a risk that this study was underpowered to detect a difference in complications. However, our post hoc sample size calculation of 373 would be nearly twice as large as any existing study on this topic and may be unattainable.

Studies investigating trainee involvement in complication rates have shown mixed findings. Caveny et al. identified 2,637 POP repair cases using the national surgical quality improvement database and found that resident involvement was associated with increased adverse outcomes (notably UTIs), length of stay, and procedure length [15]. In contrast, another study using similar methods analyzed 7,752 POP surgeries and found that although residents prolonged operative times, they did not increase complications [16]. Many other studies support that trainee involvement in urogynecological surgeries does not increase the risk of complications [10,11,14]. Most of these studies include attending surgeons with varying experiences, which may have influenced outcomes. Ultimately, complications are relatively low in our study and other studies reported in the literature for POP repairs. 

One potential limitation of this study is the inclusion of two hospitals, where surgeries at one hospital included only a PA, whereas surgeries at the other included only RES. Variability in OR staff or OR flow can impact outcomes, and there is no way to account for this in this analysis. However, with a single surgeon, similar instrumentation and surgical planning, operative time defined as skin incision to skin closure, and similar protocols under the umbrella of a single hospital system, we feel this impact was minimized.

Another limitation of this study is the inability to capture data on baseline resident skill level or the percentage of each case performed by the resident. Residents spend an entire month on urogynecology, and we would expect their surgical technique to improve with the number of cases they perform. This can also have countervailing effects on both OR time and complications. Since the learning curve of laparoscopic procedures is approximately 30 cases, changes from one case to the next can be significant [17-19]. Residents performed more aspects of the case as their skill level improved, although case difficulty, which was unable to be quantified, likely also influenced the extent of their participation.

Despite not being able to quantify these variables, case difficulty was likely similar between the RES and PA groups, and the RES group still had elevated OR times. Ultimately, four to eight-week rotations are a standard aspect of resident education, and we feel that the data exemplify the typical impact when residents assist fellowship-trained surgeons. Lastly, reproducibility and applicability to other sites may be limited due to the inclusion of a single attending surgeon and PA. However, this reduces or eliminates the impact of variations in operative technique, which can affect operative time.

The greatest strength of this work was the large volume of surgeries, with a standardized, reproducible outcome of OR time and reduced variability from a single surgeon. Manual chart review likely captured all available complications without relying on coding or billing data. Ultimately, because our findings are consistent with increases in OR time with trainees reported in other studies, it is likely our results are valid.

## Conclusions

When a fellowship-trained attending urogynecologist operated with a resident instead of a PA, operative time increased, but complications did not. Though our study concludes that resident involvement does not appear to increase complication rates, future studies may need to enroll a larger sample size to further support this conclusion. Additionally, prolonged operative times can lead to increased hospital costs due to increased staffing needs. Future studies can focus on determining which steps of the procedure prolong operative time to help identify opportunities for improving clinical instruction. Continued work is needed to balance and optimize the sometimes competing interests of both the educational component of surgical training and the quality of patient care we provide.
